# Physical, Chemical, and Bioactive Properties of Bread Made from Particle-Size-Fractionated Wheat Flour

**DOI:** 10.3390/foods15152644

**Published:** 2026-07-28

**Authors:** Samson Adeoye Oyeyinka, Oluseyi Moses Ajayi, Olayemi Eyituoyo Dudu, Jill Ellis, Oluwafemi Ayodeji Adebo

**Affiliations:** 1 Centre of Excellence in Agri-Food Technologies, National Centre for Food Manufacturing, University of Lincoln, Holbeach PE12 7PT, UK; majayi@lincoln.ac.uk (O.M.A.); jiellis@lincoln.ac.uk (J.E.); 2 Centre for Innovative Food Research (CIFR), Department of Biotechnology and Food Technology, Faculty of Science, University of Johannesburg, P.O. Box 17011, Johannesburg 2028, Gauteng, South Africa; oadebo@uj.ac.za; 3 Department of Food Science and Technology, Bells University of Technology, Ota 112104, Ogun State, Nigeria; oedudu@bellsuniversity.edu.ng

**Keywords:** wheat flour fractionation, antioxidant activity, phenolic compounds, bread quality, sensory acceptability

## Abstract

Bran enrichment improves the nutritional value of wheat-based bread but often impairs appearance, structure and sensory acceptance. This study investigated the role of controlled particle-size fractionation of brown wheat flour (BWF, equivalent to whole wheat flour) into <500 µm and <250 µm fractions in balancing bioactive enhancement and functional potential with acceptable product quality. White wheat flour (WWF) served as a refined control. Bread samples were prepared from WWF, BWF and their two bran-rich fractions, and assessed for appearance, color, physicochemical properties, texture, total phenolic content (TPC), ABTS antioxidant activity, individual phenolics, sensory attributes, correlations and chemometric patterns (using Principal Component Analysis (PCA) and Orthogonal Partial Least Square-Discriminatory Analysis (OPLS-DA)). Flour particle size strongly influenced bread color, texture and crumb structure: BWF bread samples were darkest, firmest and most chewy, whereas <250 µm bread was lighter and softer. TPC and ABTS activity were highest in BWF, followed by <500 µm and <250 µm, mirroring phenolic retention. Specifically, total phenolic content reached 1.58 mg GAE/g and ABTS antioxidant activity 19.49% in BWF, compared with 0.96 mg GAE/g and 18.31% in the <500 µm fraction, and 0.87 mg GAE/g and 17.67% in the <250 µm fraction. Bread hardness decreased from 5437 g in BWF to 4878 g in the <500 µm fraction and 4386 g in the <250 µm fraction. Key phenolic acids, chlorogenic, p-coumaric, sinapic and trans-ferulic acids, showed strong positive correlations with hardness and chewiness and negative correlations with appearance and overall acceptability. PCA and OPLS-DA confirmed distinct biochemical separation among bread types, with the PCA model explaining 73% of the total variance (PC1 = 57% and PC2 = 16%), and phenolic subclass distribution was identified as the main discriminant factor. Bread from the <500 µm fraction preserved phenolics but reduced sensory quality, while finer fractions (<250 µm) improved acceptability at the cost of some flavonoids and antioxidant capacity. Fractionation of flour, therefore, provides a practical strategy to optimize bioactive–sensory trade-offs in fiber-enriched bread samples. This study contributes to efforts in achieving sustainable development goals aimed at improving nutrition and food security.

## 1. Introduction

Bread is one of the most widely consumed staples across continents because it is affordable, widely available and represents a convenient food item [1,2]. It is predominantly made from cereal flours such as wheat, spelt and rye that contain gluten [3]. During the processing of wheat, a roller mill is used to produce flours of varying particle sizes, resulting in a range of flour types, including whole meal, brown, and white flour [4,5]. This range of flours has different characteristics which may impact the quality of the bread produced. Previous researchers also suggested that bread quality depends on several factors such as flour type [6], recipe used [7] and flour composition [8]. In general, baked products such as bread from whole-meal flour have proven to have superior health benefits than bread from white flour, as the former contains high levels of dietary fiber, vitamins and antioxidants, which support human health [4]. However, the consumption of whole wheat bread remains low across all age groups in the UK [9], presumably due to poor sensory characteristics. This challenge is not peculiar to bread alone but has been reported for other cereal-based foods such as pasta made from whole wheat [10,11,12]. Besides the sensory properties, the presence of bran in whole wheat flour often results in undesirable effects on dough rheology and bread quality, including denser texture, reduced loaf volume and altered sensory characteristics [13].

To address these challenges, fractionation of whole wheat flour into different particle-size fractions has been proposed as an effective approach to optimize bread quality while retaining its nutritional advantages [14,15]. Beyond nutritional enhancement, phenolic-rich bran fractions are known to interact with gluten proteins and influence dough structure, crumb texture, color development, and sensory perception, thereby shaping the quality trade-offs observed in whole-grain bread. However, the influence of bran particle size on bread characteristics remains inconsistent across studies. Some researchers have reported that reducing bran particle size can improve loaf volume and bread texture [16,17]. In contrast, other studies indicate that finer bran particles may increase crumb density and result in darker bread color [18,19]. These conflicting findings highlight the complex role of particle size in determining bread quality. Generally, finer flour fractions tend to promote the formation of a stronger gluten network and improve dough elasticity, which can positively influence the physical and sensory properties of baked products. For example, flat bread samples such as parotta prepared from finer flour fractions have been reported to exhibit softer textures and higher sensory acceptability [20]. Fractionation separates the bran components into fine and coarse fractions, which may influence the functional properties of flour and the final bread product differently. This approach allows better control over the balance between nutritional enrichment and product quality.

In a previous study, the bioactive composition and flour-level properties of particle-size-fractionated wheat flours were characterized [21]. Building on these findings, the present study independently evaluates how these fractions perform when applied in breadmaking, with emphasis on technological quality, sensory acceptability, and phenolic retention. Four flour samples, white wheat flour (WWF), brown wheat flour (BWF, corresponding to whole wheat flour containing bran, germ, and endosperm), and BWF fractionated through 250 µm and 500 µm sieves, were evaluated. The results showed that BWF exhibited the highest water absorption capacity, total phenolic content, total flavonoid content and antioxidant activity, while the <500 µm fraction displayed bioactive levels comparable to BWF and higher than those of the <250 µm fraction and WWF, likely due to its higher bran and fiber content. Building on these findings, this study examines the application of these fractionated flours in bread production. Specifically, this study evaluated the effect of flour fractionation at 250 µm and 500 µm on the physical, chemical, sensory and bioactive properties of leavened bread. The goal was to determine whether fractionated flour can improve bread quality while enhancing its bioactive properties relevant to nutritional quality as reflected by phenolic content and antioxidant activity, thereby supporting the development of healthier bakery products with acceptable sensory characteristics.

## 2. Materials and Methods

### 2.1. Materials and Flour Fractionation

Commercially milled wheat flours, including brown wheat flour (BWF) and white wheat flour (WWF), were obtained from Tesco Groceries in Holbeach, UK. The flours were analyzed in their original form without any additional preparation steps. For particle-size separation, batches of 250 g were processed using a stack of laboratory sieves (Endecotts Ltd., London, UK). Material passing through the 500 µm mesh was collected and designated as the <500 µm fraction, representing a coarse, bran-enriched portion of the flour. The fraction that passed through the 250 µm sieve was collected separately as the <250 µm fraction, which corresponded to a finer, more bran-rich subset of the <500 µm material. Flour fractions (<250 µm and <500 µm) were obtained directly from whole wheat flour by sieving using a stack of calibrated sieves. The particle-size fractions (<500 µm and <250 µm) were operationally defined by sieve aperture and selected to represent coarse and fine bran-rich fractions commonly used in wheat fractionation studies. Detailed study on the flour characteristics, including proximate composition of these fractions, has been reported elsewhere [21] and is not repeated here, as the present work focuses on their application in breadmaking.

All sieved fractions were transferred into airtight Ziplock^®^ polyethylene bags (Tesco, Welwyn Garden City, UK) immediately after separation and stored under ambient conditions until further analysis, dough preparation and subsequent bread analyses. The aim of this approach was to evaluate how differences in flour particle size and bran distribution influence bread quality, independently of the nutritional functionality that had been characterized previously [21]. The experimental design focused on a targeted comparison of predefined flour particle-size fractions to allow clear evaluation of particle-size effects on bread quality and bioactive properties under controlled conditions. For clarity, the flour samples are consistently referred to as WWF (white wheat flour), BWF (brown wheat flour), the <250 µm fraction (fine bran-rich fraction), and the <500 µm fraction (coarse bran-rich fraction) throughout the manuscript.

### 2.2. Production of Bread

Bread was made following a recipe developed within the food processing laboratory at the National Centre for Food Manufacturing, University of Lincoln, UK. Briefly, each flour fraction and the control samples were used to make bread following a standard straight-dough approach. The dry ingredients, 1300 g of flour, 14 g of instant yeast and 18 g of salt, were first combined. A premixed butter–sugar blend (120 g butter and 140 g sugar) was incorporated, followed by the gradual addition of 700 mL of water during low-speed mixing until a smooth, cohesive and elastic dough was formed. The dough was then manually kneaded and portioned into 600 g units. Each dough piece was allowed to proof for 1 h at 25–27 °C, after which it was divided into 150 g pieces and subjected to a second proofing period of 30 min. Baking was carried out at 160 °C for 20–25 min. All bread samples were cooled to room temperature before subsequent analysis. All bread formulations were prepared using identical ingredient ratios and water addition in order to isolate the effect of flour particle size on bread quality. Although bran-rich fractions are known to differ in water absorption capacity, hydration was not adjusted in this study to avoid confounding formulation effects. The resulting differences, therefore, reflect the combined influence of particle size and inherent flour composition under standardized processing conditions. Dough pieces were portioned to a fixed weight of 52 g in small pans prior to proofing and baking; post-baking bread weight was recorded and used to calculate baking weight loss.

### 2.3. Physical Properties of Bread

#### 2.3.1. Color

Color was determined using a bench top HunterLab colorimeter (Model A60–1014–593; Hunter Associates Laboratory, Reston, VA, USA) following the method described by Agba et al. [22]. Prior to analysis, the device was standardized using a certified white calibration tile. The color attributes were expressed in the CIE Lab* space, where L* indicates brightness (0 = black, 100 = white), a* denotes the red–green axis (positive values indicating red), and b* represents the yellow–blue axis (positive values indicating yellow). Color was measured on both the bread crust and crumb.

#### 2.3.2. Texture

Bread crumb texture was evaluated using a TA.XTplus Texture Analyser (Stable Micro Systems, Vienna Court, UK) following the method previously reported by Bilgic and Sensory [23]. Loaves were sliced into sections approximately 25 mm thick, and circular crumb samples were extracted from the central region of each slice using a 40 mm stainless steel cutter. Compression tests were performed using a 25.4 mm diameter cylindrical probe (TA11/1000, Stable Micro Systems, Vienna Court, UK). Each sample was compressed twice to 40% of its original height at a test speed of 1.7 mm s^−1^ with a trigger force of 5 g. Texture parameters, including hardness, resilience, cohesiveness, and chewiness, were calculated from the resulting force–time curves.

#### 2.3.3. Weight and Specific Volume

The weight of the bread loaves was first measured using a digital balance after cooling to room temperature. The volume of the bread samples was then determined using the rapeseed displacement method [24]. The specific volume (mL/g) of the bread was calculated as the ratio of loaf volume (mL) to loaf weight (g).

### 2.4. Analysis of Bioactive Compounds

#### 2.4.1. Total Phenolic Content

The total phenolic content of the bread samples was determined using the Folin–Ciocalteu reagent method [25]. Absorbance was measured at 765 nm using a UV–Vis spectrophotometer and quantified against a calibration curve prepared with gallic acid (Sigma-Aldrich, Dorset, UK) as the standard. The results were expressed as milligrams of gallic acid equivalents per gram of sample (mg GAE/g). It should be noted that this extraction and assay quantify the solvent-extractable phenolic fraction (free and loosely bound phenolics) and do not represent the total phenolic content that would be obtained following hydrolytic release of bound phenolics.

#### 2.4.2. ABTS

*In vitro* antioxidant activity was assessed using the ABTS radical scavenging assay described by Kewuyemi et al. [26]. The working solution was combined with the sample extract and incubated at room temperature for 30 min. Absorbance was measured at 734 nm using a spectrophotometer (Accuris Instruments, Edison, NJ, USA), and the percentage inhibition of the ABTS radical was calculated.

#### 2.4.3. Phenolic Compounds

Phenolic compounds were extracted with 80% aqueous methanol containing 1% hydrochloric acid (HCl). The mixture was vortexed (K-550-GE, Scientific Industries, Inc., Bohemia, NY, USA) and sonicated in a water bath ultrasonicator (Argolab AU-220, Carpi, Italy) for 1 h. The extract was centrifuged at 2900× *g* for 10 min at 4 °C using an centrifuge (Eppendorf 5702R, Hamburg, Germany). The resulting supernatant was filtered and used for subsequent analyses. Targeted phenolic compounds, including chlorogenic acid, trans-ferulic acid, p-coumaric acid, sinapic acid, quercetin, apigenin and luteolin, were separated and quantified using ultra-high-performance liquid chromatography with a photodiode array detector (UHPLC-PDA; Shimadzu Corporation, Kyoto, Japan). Chromatographic conditions, including column type, mobile phase composition, and gradient program, were adopted from Kewuyemi et al. [27]. Concentrations of the phenolics were expressed in micrograms per gram of sample (µg/g). Bioactive compounds were analyzed in fresh bread samples after cooling to room temperature, and results are reported on a wet weight basis.

### 2.5. Sensory Evaluation

The sensory evaluation was conducted in a dedicated sensory testing facility, with each assessor seated in an individual booth to minimize distraction and ensure independent assessment. A total of 50 untrained participants took part in the study. The test protocol was delivered using RedJade™ sensory software (RedJade, Pleasant Hill, CA, USA), which was used to construct the survey, generate four-digit randomized blind sample codes, and collect participant consent and response data. Panelists accessed the survey via a secure QR code link on their personal devices. An acceptance test was employed using a standard 9-point hedonic scale, where 1 represented “extremely dislike” and 9 represented “extremely like.” Participants evaluated each sample for appearance, aroma, texture and taste, and provided an overall acceptability rating. Water was used as a palate cleanser in between the tests.

### 2.6. Statistical Analysis

Bread samples were prepared in triplicate, and all analyses were conducted in three replicates. The resulting data were analyzed using one-way analysis of variance (ANOVA) with SPSS software (Version 21.0, IBM Corp., Armonk, NY, USA). When significant differences were detected among sample means, post hoc comparisons were performed using Duncan’s multiple range test at a 95% confidence level (*p* ≤ 0.05). To further examine underlying trends and relationships within the dataset, Pearson correlation analysis and multivariate analysis using principal component analysis (PCA) were carried out with SPSS software (Version 21.0, IBM Corp., Armonk, NY, USA) and SIMCA P software (Version 18.0, Umetrics AB, Umeå, Sweden), respectively.

## 3. Results and Discussion

### 3.1. Appearance and Objective Color of Bread

**Figure 1:**
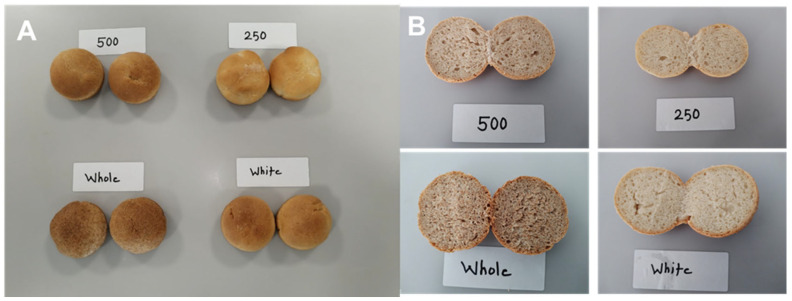
Appearance of bread from brown, white and fractionated wheat flour. White: Bread from white wheat flour (WWF); Whole: Bread from brown wheat flour, corresponding to whole wheat flour containing bran, germ, and endosperm (BWF), brown wheat flour (BWF); 500: Bread from coarse bran-rich fraction (<500 µm); 250: Bread from medium bran-rich fraction (<250 µm). (**A**) External appearance of bread rolls produced with different formulations; (**B**) Internal crumb structure of the corresponding bread rolls.

Flour particle size significantly influenced the bread characteristics, including external appearance and crumb structure, resulting in noticeable differences in crumb openness (Figure 1A,B). As shown in Figure 1A, the external appearance of the bread varied across flour types. Bread produced from brown wheat flour (BWF) appeared darker and exhibited a more compact, denser surface compared to bread made from white wheat flour (WWF) and the fractionated flours. Correspondingly, Figure 1B shows that the internal crumb of BWF bread was less open and more tightly structured, likely due to its higher bran content. A previous study on flour functionality reported that BWF contained 2–4 times more dietary fiber than the <250 µm and <500 µm fractions and WWF, a difference attributed to the presence of bran in the flour [21]. In that study, flour protein and dietary fiber contents, water absorption behaviors, and phenolic composition were systematically characterized for the same particle-size fractions; the present work focuses on translating these flour-level properties into bread quality, texture, and sensory performance.

Objective color data for both bread crust and crumb were significantly affected by flour type and particle size (Table 1). Crust lightness (L*), where higher values indicate lighter coloration, was highest in the <250 µm and WWF bread samples, corresponding to lighter crust formation, whereas the BWF sample exhibited the lowest L* values, indicating the darkest crust. This trend is consistent with the higher bran content and pigment concentration typically associated with enhanced Maillard browning in whole-grain breads.

The <500 µm sample also showed relatively low L* values, suggesting darker crust formation compared with finer fractions. Crust a* and b* values, representing the red–green and yellow–blue color axes, respectively, were generally higher for WWF and the <500 µm samples, indicating more intense red–brown and yellow color development. In contrast, the <250 µm fraction exhibited lower a* and b* values, corresponding to a milder brown crust.

Crumb color followed similar trends. WWF showed the highest crumb L* values, indicating a lighter crumb, while the <250 µm fraction also remained relatively light. The <500 µm fraction produced a darker crumb, and BWF exhibited the lowest L* values overall. Variations in crumb a* and b* values further demonstrate the influence of flour composition and particle size on internal bread color. Similar effects of bran content and particle size on bread crust and crumb color have been reported previously [19,28].

### 3.2. Weight, Weight Loss and Specific Volume of Bread

The weight, weight loss and specific volume of the bread samples were significantly affected by flour type and particle size (Table 1). Bread made from WWF was heavier than bread from BWF and the <250 µm fraction, but had a similar weight to bread from the <500 µm fraction. BWF and the bran-rich fractions (<250 µm and <500 µm) showed higher baking weight loss than refined flour bread, indicating reduced water retention during baking. Baking weight loss is a commonly used indicator of moisture retention in fiber and whole-grain breads, with higher losses frequently reported for fiber-rich formulations [29]. Previous studies have shown that bran disrupts gluten structure and baking stability, facilitating moisture escape during baking [30].

Specific volume values ranged from 2.26 to 2.71 cm^3^/g, consistent with values reported in the literature [31,32]. Specific volume is a key indicator of bread quality, influencing crumb texture and consumer preference [33]. Bran particles can physically disrupt the dough’s foam structure, reducing bread volume [30,34,35]. The effect of particle size on specific volume is not consistent across studies; some researchers have reported that finer flour fractions produce bread with higher specific volume [14], while others observed the opposite [36].

### 3.3. Texture of Bread

The textural properties of the bread samples were significantly influenced by both flour type and particle size (Table 1). Hardness ranged from 3829.78 g in WWF bread to 5436.63 g in BWF bread, indicating that bread made from brown wheat flour was the firmest, whereas those produced from WWF were the softest. The <500 µm particle-size fraction exhibited higher hardness (4878.41 g) compared with the <250 µm fraction (4385.97 g), suggesting that coarser bran particles contribute to a denser, more rigid crumb structure. This observation aligns with previous studies, which reported that larger bran particles disrupted gluten development and reduced gas retention, ultimately increasing bread firmness [35,37,38].

Resilience, a measure of the bread’s ability to recover after deformation, was highest in BWF (30.69%) and in bread made from the <250 µm fraction (29.58%). Slightly lower values were observed for WWF (26.58%) and the <500 µm fraction (25.54%). Cohesiveness followed a similar pattern: BWF bread had the highest cohesiveness (0.64), reflecting stronger internal structural bonding, while the other samples showed comparable values (0.58–0.59).

Chewiness was also markedly affected by flour characteristics. BWF bread exhibited the highest chewiness (3134.86 g), whereas WWF bread was the least chewy (2030.83 g), reinforcing the trend that higher bran content and coarser particle sizes increase the energy required for mastication. Similar increases in hardness and chewiness with coarse bran enrichment have been previously documented. The observed patterns are consistent with the findings of Ma et al. [38], who reported that bread texture becomes harder and cohesiveness decreases with higher fiber inclusion.

Overall, bread prepared from the <250 µm fraction demonstrated lower hardness and chewiness, indicative of a softer and more palatable crumb structure. In contrast, bread made from coarser flour and brown whole wheat flour was firmer and required more effort to chew.

### 3.4. Total Phenolic Content and ABTS in Bread

All values for total phenolic content, ABTS antioxidant activity, and individual phenolic compounds reported in this study refer to the solvent-extractable phenolic fraction. The TPC and ABTS radical scavenging activity of the bread samples varied significantly with flour type and particle size (Table 2). Among all samples, BWF exhibited the highest TPC (1.58 mg GAE/g), which was almost double the values observed for <250 µm (0.87 mg GAE/g), <500 µm (0.96 mg GAE/g), and WWF (0.85 mg GAE/g). The markedly higher phenolics in BWF reflect its greater bran content, as phenolic compounds, particularly trans-ferulic acid and other bound phenolics (Table 2), are predominantly concentrated in the aleurone and outer bran layers of wheat [39,40]. The trend in this study is consistent with previous reports that whole brown wheat flours contain substantially more phenolics than white or refined flours due to their higher fiber and bran fractions, although this also depends on the variety of wheat [41,42,43]. For instance, the content of bound phenolic acids in winter wheat was found to be significantly higher than that of spring wheat [41]. Furthermore, particle size seems to significantly influence the TPC values, as the <500 µm fraction recorded a significantly higher TPC than the <250 µm fraction, indicating that coarser particles retain more phenolic compounds. This may be attributed to the fact that phenolics in wheat bran are mostly bound to cell wall components; thus, larger particles preserve intact bran fragments with less disruption of their phenolic-rich structural matrices.

A similar pattern was observed for ABTS radical scavenging activity. BWF demonstrated the highest antioxidant activity (19.49%), followed by the <500 µm fraction (18.31%) and the <250 µm fraction (17.67%), while WWF showed the lowest activity (14.53%). This strong correspondence between TPC and ABTS activity confirms that phenolic compounds are major contributors to antioxidant capacity in wheat-based ingredients. The superior antioxidant activity of BWF reinforces the idea that fiber-rich bran delivers both higher phenolic loads and enhanced radical quenching properties.

The slightly higher ABTS activity in the <500 µm fraction compared with the <250 µm fraction further supports the notion that coarser bran particles provide a greater reservoir of bound phenolics, which are released during extraction and contribute to measured antioxidant capacity.

### 3.5. Phenolic Compounds in Bread

The concentrations of individual phenolic compounds differed significantly among the bread samples and closely reflected the trends observed in total phenolic content (TPC) and ABTS radical scavenging activity (Table 2). Across all measured compounds, including chlorogenic acid, trans-ferulic acid, quercetin, apigenin, p-coumaric acid, luteolin and sinapic acid, BWF consistently exhibited the highest levels compared to other samples. This compositional richness corresponds with the markedly elevated TPC and ABTS previously observed, confirming that BWF possesses the greatest antioxidant potential among the tested samples.

Phenolic acids dominated the phenolic profile of the flours, with trans-ferulic acid presenting the highest concentrations across all samples (Table 2). BWF contained 13.18 µg/g of trans-ferulic acid, more than double the amount found in <250 µm and WWF flours. Similarly, chlorogenic acid was highest in BWF (7.57 µg/g), followed by the <500 µm (5.79 µg/g) and <250 µm fractions (2.95 µg/g), with WWF showing the lowest value (1.52 µg/g). The significantly higher phenolic acid concentrations in BWF are attributed to the enrichment of bran and aleurone layers, which are the primary reservoirs of bound phenolics, particularly trans-ferulic, sinapic and p-coumaric acids [44,45]. This distribution strongly agrees with the TPC trend, indicating that phenolic acids constitute the dominant contributors to total phenolic load. The same pattern was observed in ABTS activity, reinforcing the central role of these compounds in radical scavenging.

In terms of particle size, a notable effect on the retention of phenolics was observed. The <500 µm fraction exhibited substantially higher levels of key phenolic acids, chlorogenic acid (5.79 µg/g), p-coumaric acid (1.63 µg/g) and sinapic acid (2.51 µg/g), than the finer <250 µm fraction (Table 2). This supports the notion that coarser bran particles retained more intact structural matrices, thereby preserving bound phenolic compounds more effectively than finer particles. As a result, the <500 µm sample showed higher TPC and ABTS activity than the <250 µm fraction. Similarly, the flavonoids such as quercetin, apigenin and luteolin, though present at lower concentrations, display a consistent pattern across samples (Table 2). BWF contained the highest flavonoid concentrations, particularly luteolin (0.71 µg/g) and quercetin (0.49 µg/g). The <500 µm sample again retained higher levels than the <250 µm fraction. Given the strong hydrogen-donating and radical-stabilizing capacities of flavonoids, their presence likely enhanced the overall ABTS scavenging performance of these flours. Phenolic compounds are important dietary constituents that have been widely associated with health-related effects in the literature, largely due to their antioxidant and bioactive properties [45,46]. However, the present study focuses on the distribution and relative abundance of solvent-extractable phenolic compounds in bread, rather than on bio-accessibility or direct physiological outcomes. The significant differences observed in both the quantity and profile of phenolic compounds across particle-size fractions and flour types demonstrate that targeted milling can be used to obtain ingredients with distinct bioactive and technological characteristics. Coarser fractions and BWF, which retained higher concentrations of trans-ferulic, chlorogenic, and sinapic acids as well as flavonoids such as luteolin and quercetin, exhibited higher total phenolic content and antioxidant capacity. While these compositional differences highlight the potential functional and bioactive relevance of phenolic-rich fractions, further studies incorporating digestion or bioavailability assessments would be required to substantiate specific health-related effects. Overall, controlled fractionation provides a technological approach for modulating phenolic retention and product characteristics in wheat-based foods.

### 3.6. Sensory Properties of Bread

**Figure 2:**
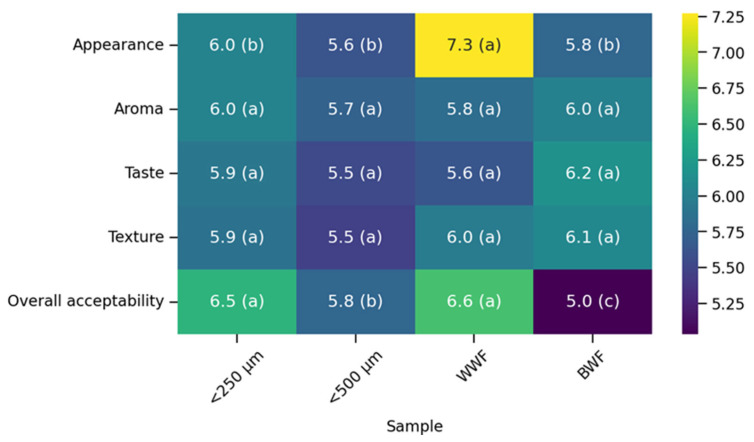
Heatmap showing the sensory characteristics of bread from brown, white and fractionated wheat flour (n = 50). WWF: Bread from white wheat flour; BWF: Bread from brown wheat flour, corresponding to whole wheat flour containing bran, germ, and endosperm; <500 µm: Bread from coarse bran-rich fraction; <250 µm: Bread from medium bran-rich fraction. Mean values with different alphabets are significantly different (*p* ≤ 0.05).

The sensory heatmap provides a visual comparison of how bran particle size and flour type affect bread quality (Figure 2). Overall, the results show that most sensory attributes were not significantly influenced by either bran particle size (<250 µm, <500 µm) or flour type (WWF, BWF) at *p* > 0.05. The only attributes that showed significant differences among samples were appearance and overall acceptability. Bread produced from WWF showed the highest rating for appearance. This observation agrees with the physical appearance of the loaves (Figure 1) and their color characteristics (Table 1), reflecting the lighter color and lower fiber load of WWF compared with BWF or the bran-rich fractions.

For overall acceptability, bread from WWF again received the highest rating, although its score was not significantly different from bread made with the finest bran particle size (<250 µm). This suggests that reducing bran particle size can help preserve or even enhance consumer acceptance in fiber-enriched bread.

Interestingly, aroma, taste and texture did not differ significantly among the samples despite variation in bran size and flour type. Although instrumental texture analysis revealed significant differences in hardness and chewiness among the bread samples, these differences were not reflected in significant sensory texture or flavor ratings. This apparent discrepancy can be attributed to the difference between instrumental measurements, which capture mechanical resistance under controlled conditions, and human oral perception, which integrates multiple dynamic factors during mastication. Moderate differences in crumb firmness that are statistically significant instrumentally may remain below the sensory discrimination threshold of untrained consumers, particularly when evaluated within a familiar food matrix such as bread. Similarly, despite measurable differences in phenolic content and composition, flavor scores did not differ significantly among samples. This may reflect the relatively low absolute concentrations of individual phenolic compounds, which, although sufficient to influence antioxidant capacity, are unlikely to impart pronounced bitterness or astringency at the levels present. In addition, flavor perception in bread is strongly influenced by Maillard reaction products and fermentation-derived volatiles, which can dominate sensory perception and reduce the impact of phenolic-related taste cues. These factors may explain why instrumental and compositional differences did not translate directly into perceptible changes in flavor or oral texture. This indicates that fiber level and particle size primarily influence visual attributes, while flavor and mouthfeel remained relatively stable across formulations. Across the dataset, bread made from the <500 µm fraction consistently yielded the lowest sensory values, appearing in the lowest-ranking significance groups. This trend highlights the potential importance of bran refinement strategies, such as re-milling or size fractionation, to improve the sensory performance of nutritionally enriched bread samples. Overall, reducing bran particle size and using refined forms of bran can mitigate sensory limitations, particularly for appearance and overall acceptability, thus improving consumer perception of high-fiber bread.

### 3.7. Pearson Correlation

The correlation analysis provides an integrated understanding of the interaction of the bioactive composition, physical properties and sensory properties across the bread samples (Table 3). TPC showed strong positive correlations with hardness, resilience, cohesiveness and chewiness (r = 0.53–0.86, *p* < 0.01). Similarly, the antioxidant activity (ABTS) showed significant positive correlation with hardness and chewiness (r = 0.68 and 0.66, *p* < 0.01). This result indicates that bread samples with higher phenolic and antioxidant levels, typically associated with greater bran inclusion, also exhibit firmer and more resistant crumb structures. These relationships suggest that bioactive enrichment through bran addition inherently modified the mechanical behavior of the dough–crumb matrix, leading to an increase in breadcrumb hardness, chewiness and structural resistance.

The individual phenolic acids showed strong positive correlations with TPC, confirming that they are major contributors to the phenolic profile of the bran-enriched bread (Table 3). Chlorogenic acid (CHA), p-coumaric acid (PCA), apigenin (APG) and sinapic acid (SNA) exhibited the highest associations with TPC (r ≈ 0.82–0.93, *p* < 0.01), indicating that variations in bran particle size and flour type directly influenced their accumulation. Similarly, all phenolic compounds showed strong and significant positive correlations with ABTS (r = 0.59–0.72 (*p* < 0.01)), which is consistent with previous studies [47,48]. These trends suggest that the antioxidant potential of the bread samples is driven by the combined and complementary contributions of these phenolic components rather than any single compound alone.

There were notable correlations between the phenolic acids and sensory properties of the bread (Table 3). Sensory appearance showed consistently inverse correlations with almost all phenolic acids (r ≈ −0.55 to −0.79, *p* < 0.01) and texture-related variables (r ≈ −0.62 to −0.71, *p* < 0.01). This aligns with the observed darker color and denser structure of high-bran bread samples, underscoring that increasing bran content, even when nutritionally advantageous, compromises visual quality. These patterns support earlier findings in the study showing that bread samples produced from WWF or fine flour (<250 µm) achieved superior appearance scores (Figure 2), consistent with their lighter color (Table 1) and more uniform crumb (Figure 1). Furthermore, TFA, APG and SNA had positive correlations with taste (r ≈ 0.57 to 0.69, *p* < 0.01). Overall sensory acceptability exhibited strong negative correlations with all phenolic compounds (−0.73 to −0.95, *p* < 0.01) and texture variables (r ≈ −0.67 to −0.87, *p* < 0.01) apart from resilience. The observed correlations between phenolic content, textural parameters, and sensory responses could be due to the combined effects of bran-associated phenolics on gluten network formation, crumb structure, and Maillard-driven color development. These relationships reflect the dual role of bran phenolics as contributors to antioxidant capacity and as structural modifiers that stiffen the dough–crumb matrix through protein binding and fiber reinforcement.

This pattern confirms that consumer acceptance was primarily influenced by structural and visual changes driven by bran content, rather than by flavor attributes. Bread from the <500 µm fraction, which consistently scored lowest in sensory evaluation, also aligned with the most unfavorable combinations of phenolic, textural, and appearance markers. These relationships support the broader conclusion that bran refinement strategies, such as re-milling or fine fractionation, can enhance the sensory performance of nutritionally enriched bread samples by reducing the mechanical penalties and appearance challenges typically introduced by bran. An earlier study also reported that phenolic compounds exhibited a negative correlation with the sensory properties of bread [49], likely due to interactions between phenolic acids and Maillard reaction adducts [50]. This mechanism may explain why bread produced from BWF and the <500 µm fraction received lower ratings for appearance and overall acceptability (Figure 2).

### 3.8. Multivariate Chemometric Analysis

**Figure 3:**
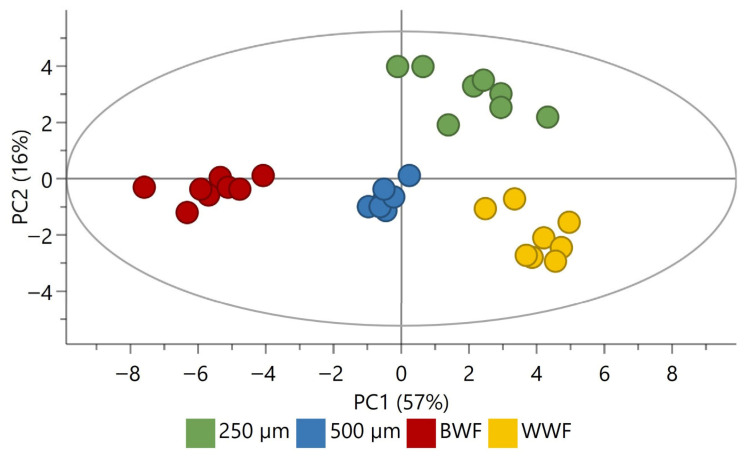
Principal component analysis of the plot of bread from brown, white and fractionated wheat flour. WWF: Bread from white wheat flour; BWF: Bread from brown wheat flour, corresponding to whole wheat flour containing bran, germ, and endosperm; <500 µm: Bread from coarse bran-rich fraction; <250 µm: Bread from medium bran-rich fraction.

A multivariate chemometric approach was applied to visualize the biochemical differentiation among bread samples produced from WWF, BWF and the two bran-rich particle-size fractions (<500 µm and <250 µm). The analyses consistently demonstrated that phenolic composition is the primary driver of variation across the bread types. The PCA of the full dataset explained 73% of the total variation (PC1 = 57%, PC2 = 16%) and revealed four well-defined clusters corresponding to the flour sources (Figure 3). The cumulative variance explained by the first two principal components (73%) is considered sufficient for reliable data interpretation in complex food systems, particularly when supported by complementary correlation analysis and supervised OPLS-DA modelling.

PC1 represented a clear phenolic–antioxidant gradient, with WWF positioned on the far right and BWF on the extreme left. The <500 µm and <250 µm fractions clustered between these extremes, reflecting intermediate phenolic abundance and antioxidant capacity. PC2 further discriminated the two bran-rich fractions, indicating differences in the distribution of specific phenolic subclasses or other functional traits influenced by particle-size reduction. Thus, these patterns confirm that controlled fractionation produces compositionally distinct bread samples.

**Figure 4:**
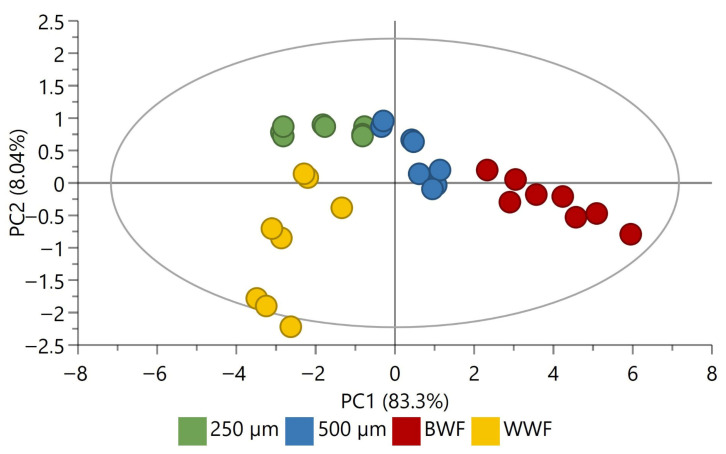
Principal component analysis of phenolic compounds of bread from brown, white and fractionated wheat flour. WWF: Bread from white wheat flour; BWF: Bread from brown wheat flour, corresponding to whole wheat flour containing bran, germ, and endosperm; <500 µm: Bread from coarse bran-rich fraction; <250 µm: Bread from medium bran-rich fraction.

To understand the specific contribution of phenolics, a second PCA was performed on phenolic compounds alone (Figure 4). The separation became more pronounced, with 91.3% of variation explained (PC1 = 83.3%, PC2 = 8.0%). PC1 clearly segregated bread samples by total phenolic content and phenolic subclass composition. BWF exhibited the highest scores, followed by the <500 µm fraction, confirming strong retention of bran-associated phenolics in these samples. The <250 µm fraction formed a distinct cluster with moderate PC1 values, consistent with its reduced TPC and ABTS antioxidant activity (Table 2). WWF remained isolated on the far left, reflecting its inherently low phenolic profile. These results demonstrate that phenolic concentration and subclass distribution dominate the multivariate differentiation of the bread samples.

To identify the specific phenolic markers driving these separations, OPLS-DA was performed (Table 4). OPLS-DA was applied as an exploratory tool to assist in visualizing discriminatory patterns among bread samples rather than as a predictive classification model. Interpretation of OPLS-DA outputs was therefore restricted to variable importance trends and was corroborated by unsupervised PCA and correlation analysis. Formal predictive validation metrics were not applied, and the results are not interpreted in a confirmatory modelling sense. The resulting *p*-values and fold-changes revealed substantial biochemical alterations among WWF, BWF and the two fractions. BWF displayed the greatest enrichment, with chlorogenic acid (CAB) and apigenin (APB) increasing nearly five-fold and four-fold, respectively. Such large increases reflect the natural concentration of hydroxycinnamic acids and bran-associated flavonoids in the aleurone and pericarp layers of whole brown wheat, consistent with established bran phytochemistry [43,51,52].

The <500 µm fraction also showed strong enrichment of phenolic acids (1.8–3.8-fold) and flavonoids (2.2–2.3-fold). This indicates that medium-coarse bran particles efficiently retain bioactive compounds during fractionation, yielding a biochemical profile closely resembling whole BWF. The associated increases in TPC and ABTS (Table 2) further confirm the strong antioxidant potential of this fraction. In contrast, the <250 µm fraction exhibited a different pattern: while several phenolic acids (chlorogenic, p-coumaric and sinapic acid) increased relative to WWF, the flavonoid quercetin decreased sharply (0.34-fold). This selective reduction suggests that fine sieving removes larger bran fragments rich in bound flavonoids, favoring the passage of endosperm-dense, phenolic-poor particles. This interpretation aligns with the phenolic-only PCA, where the <250 µm cluster occupies a distinct region separate from both <500 µm and BWF.

In all comparisons, elevated phenolic levels were accompanied by increases in ABTS antioxidant activity. The consistent positive fold-change in ABTS (1.22–1.34) supports the strong correlations observed between phenolic acids and radical scavenging capacity (Table 3). These biochemical differences have sensory implications. Higher phenolic content in BWF and <500 µm bread samples corresponds to their darker crumb and lower sensory ratings (Figure 2), likely due to phenolic–protein interactions, intensified Maillard browning, and increased bitterness. The <250 µm fraction, with intermediate phenolic enrichment and reduced flavonoids, displayed intermediate sensory performance.

**Figure 5:**
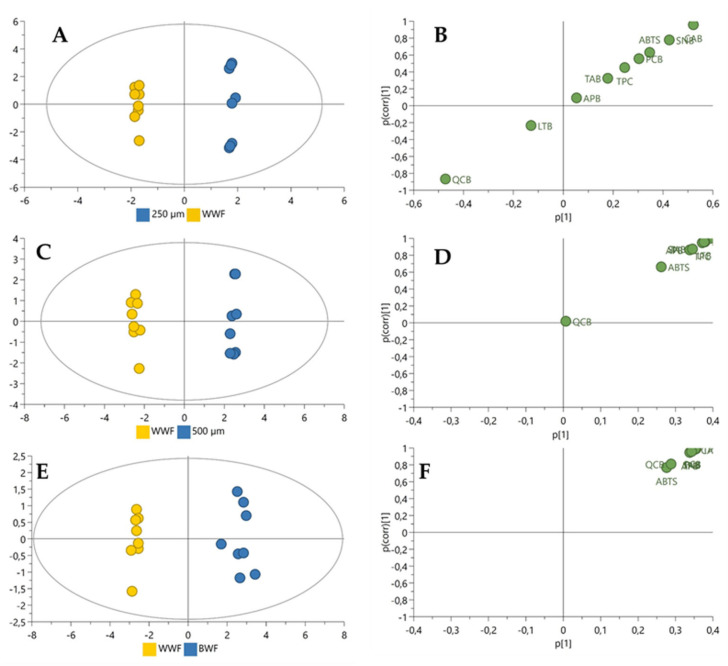
Principal component analysis and loading plots displaying discriminant total phenolics, phenolic compounds and ABTS antioxidant properties. WWF: Bread from white wheat flour; BWF: Bread from brown wheat flour, corresponding to whole wheat flour containing bran, germ, and endosperm; <500 µm: Bread from coarse bran-rich fraction; <250 µm: Bread from medium bran-rich fraction. Note: As generated by the software: (-) means – (minus sign); ‘,’ means ‘.’ (decimal). (**A**) Score plot differentiating 250 µm from WWF; (**B**) S-plot showing parameters contributing to differences between 250 µm and WWF; (**C**) Score plot differentiating 500 µm from WWF; (**D**) S-plot showing parameters contributing to differences between 500 µm and WWF; (**E**) Score plot differentiating BWF from WWF; (**F**) S-plot showing parameters contributing to differences between 250 µm and WWF.

To further visualize the discriminant metabolites identified in the OPLS-DA analysis, pairwise PCA score and S-plots were generated (Figure 5). The score plots (Figure 5A,C,E) show clear separation between WWF and each bran-rich fraction, confirming the OPLS-DA patterns. The corresponding S-plots (Figure 5B,D,F) indicate that p-coumaric acid, trans-ferulic acid, chlorogenic acid, sinapic acid and total phenolic content cluster closely with ABTS, confirming their key role in driving antioxidant differences. Quercetin loads in the negative direction specifically in the <250 µm comparison, reaffirming its selective reduction during fine sieving. Overall, the combined PCA–OPLS-DA results show that controlled fractionation modulated phenolic composition in predictable, particle-size-dependent ways, with the <500 µm fraction closely resembling BWF and the <250 µm fraction forming a unique, compositionally distinct subgroup.

## 4. Conclusions

This study demonstrated that controlled particle-size fractionation is an effective tool for modulating the bioactive, functional and sensory properties of bread enriched with brown wheat flour. Coarser bran-rich fractions (<500 µm) retained the highest levels of phenolic acids and flavonoids, resulting in elevated total phenolic content and antioxidant activity. However, these fractions also produced darker, firmer and less acceptable bread samples due to their stronger influence on crumb structure and Maillard-driven browning. In contrast, finer bran fractions (<250 µm) reduced the negative effects on appearance, crumb hardness and overall acceptability, although with a trade-off in phenolic retention, particularly for flavonoids such as quercetin. Multivariate analyses confirmed that phenolic subclass distribution is the primary driver of biochemical variation among bread types, with BWF and the <500 µm fraction clustering together due to their shared phenolic richness. Correlation analysis further revealed that phenolic enrichment is closely associated with increased hardness, chewiness and reduced sensory scores, highlighting the intrinsic tension between nutritional enhancement and consumer appeal. Overall, the findings revealed that controlled bran fractionation allows targeted improvement of bread formulations by tailoring particle size to optimize bioactive enrichment and sensory performance. This approach offers practical opportunities for the development of fiber-enriched and phenolic-enhanced wheat products with improved consumer acceptance. This will, in turn, enhance global nutrition and food security efforts, thereby supporting Sustainable Development Goal 3 (Good Health and Well-Being) through the development of foods rich in bioactive compounds with improved consumer acceptance. Future studies could build on this work by optimizing water addition based on flour-specific absorption capacity to further disentangle formulation effects from intrinsic particle-size influences.

## Figures and Tables

**Table 1:** Physical properties of bread from brown, white and fractionated wheat flour.

Parameters	<250 µm	<500 µm	WWF	BWF
L* (Crust)	62.65 ± 3.61 ^a^	52.31 ± 1.87 ^b^	61.18 ± 3.29 ^a^	49.18 ± 0.57 ^c^
a* (Crust)	8.90 ± 2.95 ^b^	15.18 ± 0.79 ^a^	15.11 ± 0.39 ^a^	14.57 ± 0.16 ^a^
b* (Crust)	22.13 ± 6.23 ^b^	22.68 ± 1.12 ^b^	26.05 ± 0.69 ^a^	17.47 ± 1.03 ^c^
L* (Crumb)	71.82 ± 2.03 ^b^	69.19 ± 0.49 ^c^	75.93 ± 2.09 ^a^	62.15 ± 1.04 ^d^
a* (Crumb)	1.82 ± 0.31 ^d^	4.31 ± 0.16 ^b^	3.10 ± 1.71 ^c^	6.84 ± 0.16 ^a^
b* (Crumb)	16.72 ± 0.34 ^b,c^	17.09 ± 0.11 ^b^	18.78 ± 1.74 ^a^	15.83 ± 0.06 ^c^
Hardness (g)	4385.97 ± 560.98 ^c^	4878.41 ± 206.22 ^b^	3829.78 ± 218.01 ^d^	5436.63 ± 503.28 ^a^
Resilience	29.58 ± 2.62 ^a^	25.54 ± 0.93 ^b^	26.58 ± 0.75 ^b^	30.69 ± 1.05 ^a^
Cohesiveness	0.58 ± 0.02 ^b^	0.58 ± 0.02 ^b^	0.59 ± 0.01 ^b^	0.64 ± 0.01 ^a^
Chewiness (g)	2258.66 ± 331.44 ^c^	2521.59 ± 169.84 ^b^	2030.83 ± 130.69 ^c^	3134.86 ± 230.98 ^a^
Specific volume (cm^3^/g)	2.71 ± 0.06 ^a^	2.26 ± 0.08 ^b^	2.35 ± 0.23 ^b^	2.36 ± 0.15 ^b^
Weight (g)	44.11 ± 0.49 ^b^	45.73 ± 0.48 ^a^	46.12 ± 0.10 ^a^	44.16 ± 0.77 ^b^
Baking loss (%)	15.17%	12.06%	11.31%	15.08%

Values are reported as mean ± standard deviation. Means with different superscripts within the same row are significantly different (*p* < 0.05). WWF: Bread from white wheat flour; BWF: Bread from brown wheat flour, corresponding to whole wheat flour containing bran, germ, and endosperm; Bread from brown wheat flour; <500 µm: Bread from coarse bran-rich fraction; <250 µm: Bread from medium bran-rich fraction. L* indicates brightness (0 = black, 100 = white), a* denotes the red–green axis (positive values indicating red), and b* represents the yellow–blue axis (positive values indicating yellow).

**Table 2:** Total phenolic content, ABTS activity and phenolic compounds of bread from brown, white and fractionated wheat flour.

Parameters	<250 µm	<500 µm	WWF	BWF
Total phenolic content (mgGAE/g)	0.87 ± 0.02 ^c^	0.96 ± 0.02 ^b^	0.85 ± 0.02 ^c^	1.58 ± 0.11 ^a^
ABTS (%)	17.67 ± 0.22 ^b^	18.31 ± 0.44 ^a,b^	14.53 ± 3.10 ^c^	19.49 ± 0.06 ^a^
Chlorogenic acid (µg/g)	2.95 ± 0.31 ^c^	5.79 ± 0.18 ^b^	1.52 ± 0.14 ^d^	7.57 ± 1.19 ^a^
Trans-ferulic acid (µg/g)	6.14 ± 3.09 ^c^	8.50 ± 1.09 ^b^	4.64 ± 1.22 ^c^	13.18 ± 1.65 ^a^
Quercetin (µg/g)	0.12 ± 0.01 ^c^	0.28 ± 0.12 ^b^	0.28 ± 0.07 ^b^	0.49 ± 0.11 ^a^
Apigenin (µg/g)	0.13 ± 0.06 ^c^	0.26 ± 0.06 ^b^	0.12 ± 0.03 ^c^	0.48 ± 0.09 ^a^
p-coumaric acid (µg/g)	0.83 ± 0.31 ^c^	1.63 ± 0.22 ^b^	0.55 ± 0.02 ^d^	2.15 ± 0.37 ^a^
Luteolin (µg/g)	0.19 ± 0.09 ^c^	0.49 ± 0.05 ^b^	0.22 ± 0.05 ^c^	0.71 ± 0.07 ^a^
Sinapic acid (µg/g)	2.31 ± 0.45 ^b^	2.51 ± 0.32 ^b^	1.38 ± 0.36 ^c^	3.89 ± 0.49 ^a^

Values are reported as mean ± standard deviation. Means with different superscripts are significantly different (*p* < 0.05). WWF: Bread from white wheat flour; BWF: Bread from brown wheat flour, corresponding to whole wheat flour containing bran, germ, and endosperm; <500 µm: Bread from coarse bran-rich fraction; <250 µm: Bread from medium bran-rich fraction; ABTS: 2,2′-Azinobis (3-ethylbenzothiazoline-6-sulfonic acid).

**Table 3:** Pearson correlation of bioactive, physical and sensory properties of bread from brown, white and fractionated wheat flour.

	TPC	ABTS	Hard	Res	Coh	Che	CHA	TFA	QCT	APG	PCA	LTN	SNA	App	Tex	Aro	Tas	OA
TPC	1																	
ABTS	0.55 **	1																
Hard	0.74 **	0.68 **	1															
Res	0.53 **	0.36 *	0.35	1														
Coh	0.79 **	0.35 *	0.48 **	0.72 **	1													
Che	0.86 **	0.66 **	0.96 **	0.51 **	0.71 **	1												
CHA	0.82 **	0.69 **	0.83 **	0.29	0.56 **	0.85 **	1											
TFA	0.85 **	0.63 **	0.74 **	0.49 **	0.69 **	0.82 **	0.88 **	1										
QCT	0.78 **	0.35 *	0.53 *	0.21	0.65 **	0.64 **	0.66 **	0.75 **	1									
APG	0.90 **	0.59 **	0.76 **	0.41 *	0.70 **	0.84 **	0.91 **	0.96 **	0.87 **	1								
PCA	0.811 **	0.65 **	0.79 **	0.31	0.57 **	0.83 **	0.97 **	0.94 **	0.74 **	0.96 **	1							
LTN	0.86 **	0.59 **	0.75 **	0.31	0.69	0.83 **	0.93 **	0.90 **	0.73 **	0.91 **	0.92 **	1						
SNA	0.86 **	0.72 **	0.78 **	0.57 **	065 **	0.84 **	0.89 **	0.96 **	0.70 **	0.94 **	0.92 **	0.84 **	1					
App	−0.45 **	−0.74 **	−0.71 **	−0.22	−0.16	−0.62 **	−0.79 **	−0.60 **	−0.17	−0.55 **	−0.72 **	−0.59 **	−0.69 **	1				
Tex	0.46 **	−0.09	−0.02	0.62 **	0.55 **	0.18	−0.08	0.16	0.31	0.19	−0.05	0.05	0.20	0.40 *	1			
Aro	0.38	0.26	0.18	0.79 **	0.34	0.26	0.07	0.22	−0.01	0.17	0.05	−0.01	0.39 *	−0.14	0.72 **	1		
Tas	0.75 **	0.44 *	0.47 **	0.82 **	0.64 **	0.59 **	0.45	0.57 **	0.38 *	0.56 **	0.43 *	0.42 *	0.69 **	−0.31	0.74 **	0.89 **	1	
OA	0.91 **	0.65 **	−0.81 **	−0.33	−0.67 **	−0.87 **	−0.95 **	−0.86 **	−0.73 **	−0.91 **	−0.92 **	−0.95 **	−0.86 **	0.69 **	−0.08	−0.11	−0.53 **	1

** Correlation is significant at the 0.01 level and * Correlation is significant at the 0.05 level. CHA: Chlorogenic acid; TFA: Trans-ferulic acid; QCT: Quercetin; APG: Apigenin; PCA: p-coumaric acid; LTN: Luteolin; SNA: Sinapic acid; TPC: Total phenolic content; ABTS: 2,2′-Azinobis(3-ethylbenzothiazoline-6-sulfonic acid); Hard: Hardness; Res: Resilience; Coh: Cohesiveness; Che: Chewiness; App: Appearance; Tex: Texture; Aro: Aroma; Tas; Taste; OA: Overall Acceptability.

**Table 4:** Differential phenolic compounds, total phenolics, and antioxidant activity in bread samples from fractionated wheat flours compared with white wheat flour (WWF), based on OPLS-DA *p*-values and fold-changes.

Parameter	Parameter Group	*p*-Value	Fold Change
WWF vs. <250 µm			
ABTS	Antioxidant	0.013	1.22
Chlorogenic acid	Phenolic acid	9.25 × 10^−9^	1.94
Quercetin	Flavonoid	1.30 × 10^−5^	0.34
p-coumaric acid	Phenolic acid	0.026	1.50
Sinapic acid	Phenolic acid	4.21 × 10^−4^	1.68
WWF vs. <500			
Total phenolic content	Total phenols	1.28 × 10^−7^	1.12
ABTS	Antioxidant	4.21 × 10^−3^	1.26
Chlorogenic acid	Phenolic acid	1.65 × 10^−17^	3.81
Trans-ferulic acid	Phenolic acid	1.04 × 10^−5^	1.83
Apigenin	Flavonoid	2.01 × 10^−5^	2.22
p-coumaric acid	Phenolic acid	1.33 × 10^−9^	2.95
Luteolin	Flavonoid	3.08 × 10^−8^	2.24
Sinapic acid	Phenolic acid	1.22 × 10^−5^	1.82
WWF vs. BWF			
Total phenolic content	Total phenols	1.94 ×10^−11^	1.85
ABTS	Antioxidant	4.87 × 10^−4^	1.34
Chlorogenic acid	Phenolic acid	9.86 × 10^−10^	4.98
Trans-ferulic acid	Phenolic acid	1.17 × 10^−8^	2.84
Quercetin	Flavonoid	2.21 × 10^−4^	1.80
Apigenin	Flavonoid	9.63 × 10^−8^	4.10
p-coumaric acid	Phenolic acid	6.51 × 10^−9^	3.89
Luteolin	Flavonoid	1.07 × 10^−10^	3.25
Sinapic acid	Phenolic acid	1.28 × 10^−8^	2.82

Values are reported as mean ± standard deviation. Means with different superscripts are significantly different (*p* < 0.05). WWF: Bread from white wheat flour; BWF: Bread from brown wheat flour, corresponding to whole wheat flour containing bran, germ, and endosperm; <500 µm: Bread from coarse bran-rich fraction; <250 µm: Bread from medium bran-rich fraction.

## Data Availability

The datasets used and/or analyzed during the current study are available from the corresponding author on reasonable request.
